# Accounting for Ecosystem Alteration Doubles Estimates of Conservation Risk in the Conterminous United States

**DOI:** 10.1371/journal.pone.0023002

**Published:** 2011-08-05

**Authors:** Randy Swaty, Kori Blankenship, Sarah Hagen, Joseph Fargione, Jim Smith, Jeannie Patton

**Affiliations:** 1 The Nature Conservancy, Marquette, Michigan, United States of America; 2 The Nature Conservancy, Bend, Oregon, United States of America; 3 The Nature Conservancy, Minneapolis, Minnesota, United States of America; 4 The Nature Conservancy, Jacksonville, Florida, United States of America; 5 The Nature Conservancy, Boulder, Colorado, United States of America; Dalhousie University, Canada

## Abstract

Previous national and global conservation assessments have relied on habitat conversion data to quantify conservation risk. However, in addition to habitat conversion to crop production or urban uses, ecosystem alteration (e.g., from logging, conversion to plantations, biological invasion, or fire suppression) is a large source of conservation risk. We add data quantifying ecosystem alteration on unconverted lands to arrive at a more accurate depiction of conservation risk for the conterminous United States. We quantify ecosystem alteration using a recent national assessment based on remote sensing of current vegetation compared with modeled reference natural vegetation conditions. Highly altered (but not converted) ecosystems comprise 23% of the conterminous United States, such that the number of critically endangered ecoregions in the United States is 156% higher than when calculated using habitat conversion data alone. Increased attention to natural resource management will be essential to address widespread ecosystem alteration and reduce conservation risk.

## Introduction

Conservation assessments at regional, national, and global levels have commonly relied upon data on the magnitude and rate of habitat conversion to crop production or urban uses as an evaluation of conservation risk [1], [2], [3], [4], [5], [6]. While this approach provides useful information, it neglects the fact that much habitat — while not converted outright— could be highly degraded due to logging, fire suppression, biological invasions, grazing, and other land management practices.

Data to assess the extent of ecosystem alteration have previously not been available at broad scales. Recently, however, a national land-cover assessment of ecosystem alteration based on remote sensing and departure from reference natural vegetation conditions has been conducted for the United States (www.landfire.gov) [7], [8]. These data capture human alteration of ecosystem structure and composition through disturbances such as fire suppression, conversion to plantations, logging, and biological invasions from introduced plant species. In many cases, this altered vegetation has reduced habitat value for species of conservation concern [9], [10], [11]. For example, vegetation structure and composition affect habitat use by grassland birds [12], forest mammal diversity [13], [14], grassland arthropod diversity [15], [16], and ecosystem services [17], [18]. Therefore, conservation risk assessments must consider ecosystem alteration in addition to habitat conversion in order to fully capture impacts to biodiversity and ecosystem services.

We used LANDFIRE's national map of ecosystem alteration to calculate a conservation risk index for ecoregions in the conterminous United States, expanding a previous assessment based on habitat conversion [19]. We selected ecoregions as the scale of analysis because these geographic units share similar species, ecological dynamics, and environmental conditions and are widely used for conservation planning [20], [21].

This analysis provides, for the first time, a comprehensive picture of ecosystem alteration in the United States. Large-scale conservation planning has focused on protecting land from conversion in part because it is relatively easy to map protected and converted areas. Although management practices and associated ecosystem alteration on unconverted lands is arguably of equal or greater importance for conservation, data availability has, until now, limited consideration of ecosystem alteration in large-scale conservation planning.

## Materials and Methods

**Figure 1:**
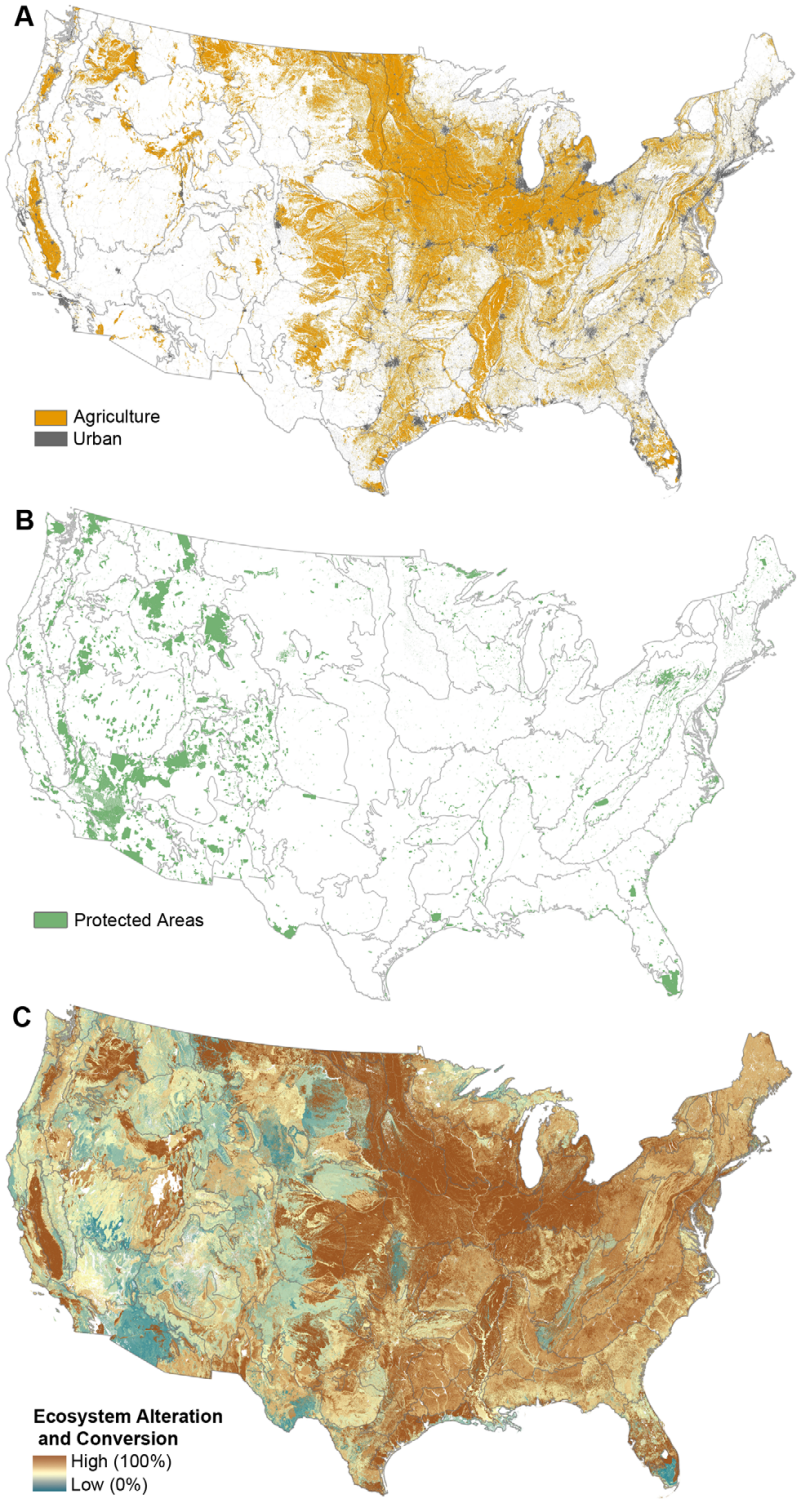
Mapping components of the ecological conservation risk index. (A) Areas converted to agricultural and urban land use, and (B) protected areas, and (C) ecosystem alteration and conversion (converted lands are considered to be 100% altered). High alteration indicates a substantial shift in vegetation structure and/or composition from reference conditions. Grey lines indicate ecoregional boundaries.

Ecosystem alteration and land conversion were assessed for the conterminous United States using LANDFIRE National Project spatial data (www.landfire.gov). LANDFIRE's measure of ecosystem alteration assesses the difference between estimated reference conditions (historic vegetation structure and composition) and current vegetation [7], [8]. Lands classified as urban, agricultural, or barren (Fig. 1A) were excluded in the LANDFIRE analysis. To generate reference conditions that incorporated natural disturbance regimes (e.g. fire, insects, and storms), LANDFIRE used the Vegetation Dynamics Development Tool (VDDT, www.essa.com) and the LANDSUM model [22], [23], [24] to estimate reference conditions within each of 1,667 Biophysical Settings (BpS; represents dominant vegetation prior to Euro-American settlement based on edaphic and disturbance factors [8]). These models, which were tailored for each BpS, predict the average proportion of an ecosystem in each of several (up to five) successional states defined by cover, height, and dominant vegetation. For example, in the Western Cascades Western Hemlock Forest, five successional states were defined as shown in Table 1. All reference vegetation for each BpS was assumed to fall into one of the defined successional states.

**Table 1:** Definitions for the succession classes in the Western Cascades Western Hemlock Forest.

Succession Class	Vegetation Height	Vegetation Cover	Dominant Vegetation
A	<5 meters	0–60%	fireweed (*Epilobium angustifolium*) and red alder (*Alnus rubra*) with tree seedlings
B	5–50 meters	61–100%	Douglas-fir (*Pseudotsuga* menziesii) and western hemlock (*Tsuga heterophylla*)
C	5–50 meters	20–60%	Douglas-fir (*P. menziesii*) with some shrubs such as salal (*Gaultheria shallon*)
D	>50 meters	20–60%	Douglas-fir (*P. menziesii*)
E	>50 meters	61–100%	Douglas-fir (*P. menziesii*) and Western hemlock (*T. heterophylla*)

Any particular location is expected to transition through successional states over time. Given this dynamic nature of vegetation, it is not possible to assign any particular location to a single reference successional state. Therefore, reference vegetation models were designed to predict the proportion of land cover in different successional classes for the entire extent of a BpS rather than to make fine-grained predictions about land cover.

To map current vegetation type, cover, and height, each 30-meter pixel in the United States was categorized based on remotely sensed data trained using 331,900 ground-truth vegetation plots [8]. The classification system recognized 398 existing vegetation types, 27 cover classes, and 12 height classes. Current land cover was categorized based on the same successional states defined in the reference vegetation analysis, using the three current vegetation data layers (i.e. vegetation type, cover, and height). Current vegetation that did not fall into one of the successional state categories was assigned to one of two alternative “uncharacteristic” states: uncharacteristic native or uncharacteristic exotic. Although one component of the land cover data underwent validation analysis (existing vegetation type [25], [26]), the “current vegetation successional state” data layer that we used in our analysis did not.

The degree of ecosystem alteration in each ecosystem was quantified using a similarity index based on the proportion of land cover in different successional states in reference versus current conditions [8]. An alteration metric was computed for each BpS in each Ecological Subsection (hereafter “ecosystems”) [27]. This ecosystem alteration index ranges from 0 to 100 (Fig. 1C), with scores of 67 and higher considered to indicate highly altered ecosystems [28]. To assess the sensitivity of our results to this threshold, we also calculated our results using a threshold of 57 and 77. The use of this threshold acts to exclude areas that are not highly altered from subsequent analyses. We note that lands that are not “highly altered” may still be moderately altered and that this alteration may still have detrimental effects on habitat values, wildlife, and ecosystem services. If so, our assessment of conservation risk is conservative. To assess conservation risk at the scale of ecoregions (each of which contains numerous ecosystems), we tabulated the percentage of land covered by ecosystems found to be highly altered within each ecoregion.

As an index of the relative conservation risk at the ecoregional scale, we developed the Ecological Conservation Risk Index (ECRI). The ECRI is an extension of the Conservation Risk Index (CRI), which is calculated as the ratio of percent area converted to percent area protected (Fig. 1B) for a given biome or ecoregion [19]. Although other approaches are available for determining conservation risk for ecosystems [1], [4], [6], CRI is unique in that it was developed to be applied to ecoregions and the data requirements for its calculation are available at national scales. Because ecosystem alteration may also erode habitat value, we add the percent area highly altered to the percent area converted to calculate ECRI, given by the formula:
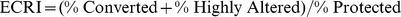



As a comparison, we applied both the CRI and the ECRI to Bailey's ecoregions [29], [30] in the conterminous United States. For CRI (or ECRI), ecoregions in which habitat conversion (and high alteration) >20% and CRI (or ECRI) >2 were classified as Vulnerable; those in which conversion (and high alteration) >40% and CRI (or ECRI) >10 were classified as Endangered; and those with conversion (and high alteration) >50% and CRI (or ECRI) >25 were classified as Critically Endangered [19].

Protected Areas were based on the 2009 World Database on Protected Areas (Fig. 1B; [31]). We included both areas designated for biodiversity protection (IUCN categories I–IV, including U.S. National Parks and Wilderness areas) and those designated for multiple management objectives (IUCN categories V–VI) in our analysis. Proposed areas, areas mapped with a point location (whose area could not be calculated), and areas or portions of areas in water were excluded from the analysis.

## Results

Approximately 29% of the land area of the conterminous United States has been converted to human use, with roughly 24% (182 million hectares) converted to agriculture and 5% (37 million hectares) converted to urban land use [7]. However, these numbers do not include the widespread occurrence of ecosystem alteration. Our analysis shows an additional 23% of non-converted lands in the conterminous United States have high levels of ecosystem alteration, indicating a significant shift in vegetation structure and composition relative to reference conditions. In total, more than half (52%) of the United States has been highly altered or converted.

**Figure 2:**
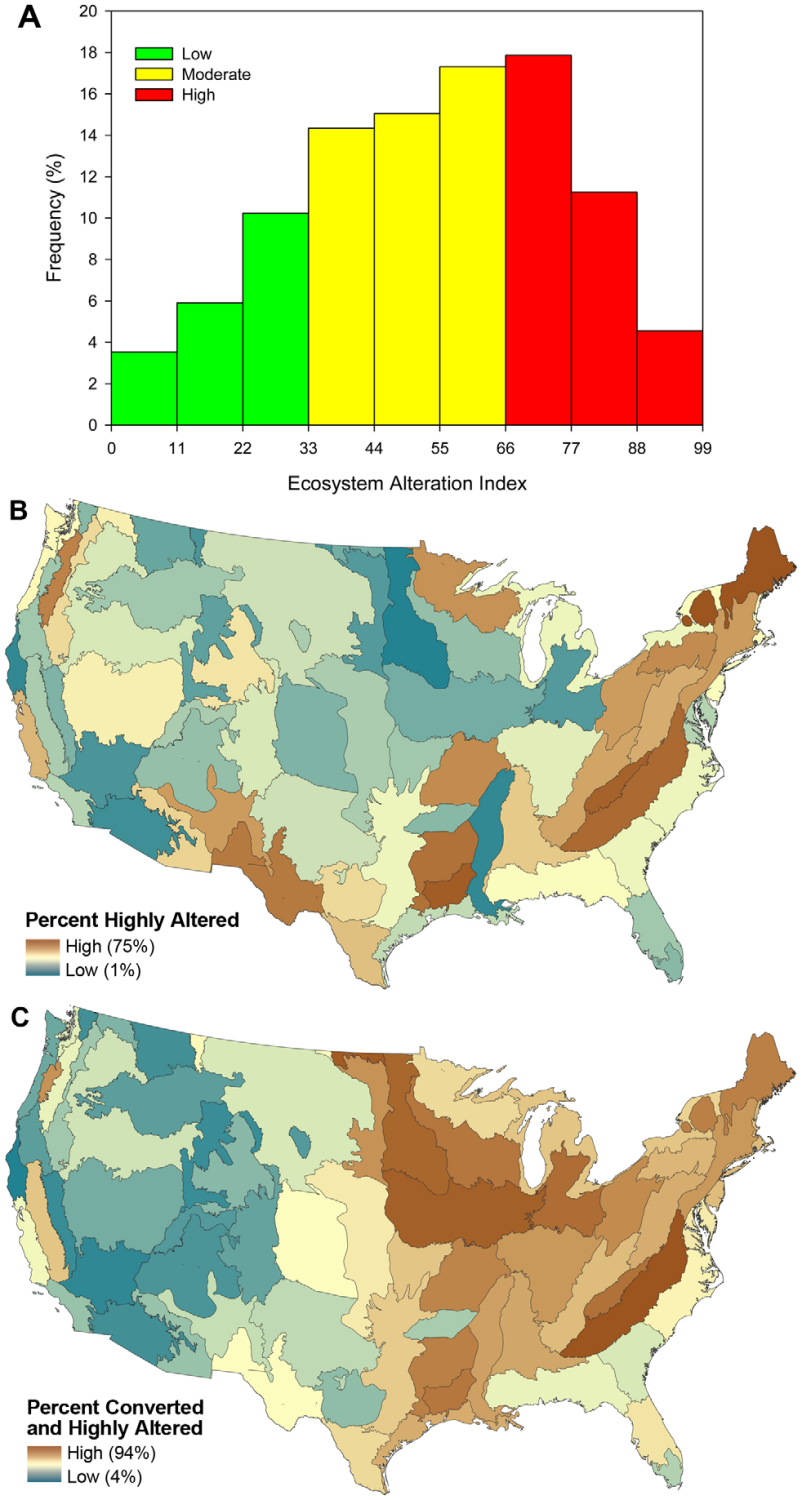
Percent highly altered and converted by ecoregion. (A) The frequency distribution of the ecosystem alteration index in the conterminous United States (excluding converted and barren lands). (B) Percent highly altered by ecoregion. (C) Percent highly altered or converted by ecoregion. Grey lines indicate ecoregional boundaries.

In addition to these highly altered lands, many lands are moderately altered. Figure 2 shows the frequency distribution for our ecosystem alteration index on unconverted lands, showing the percent of lands that have low, moderate, and high ecosystem alteration. The average ecosystem alteration index value was 54% (Fig. 2A). However, this alteration was not distributed evenly across the United States, with some areas having a higher percentage of highly altered areas (Fig. 2B). The percent of an ecoregion that was highly altered ranged from a low of 1% in the Northern Tallgrass Prairie to a high of 75% in the Northern Appalachians. When considering both ecosystem conversion and alteration, the percent of an ecoregion that was impacted ranged from 3% in the California North Coast to 94% in the Piedmont (Fig. 2C).

**Figure 3:**
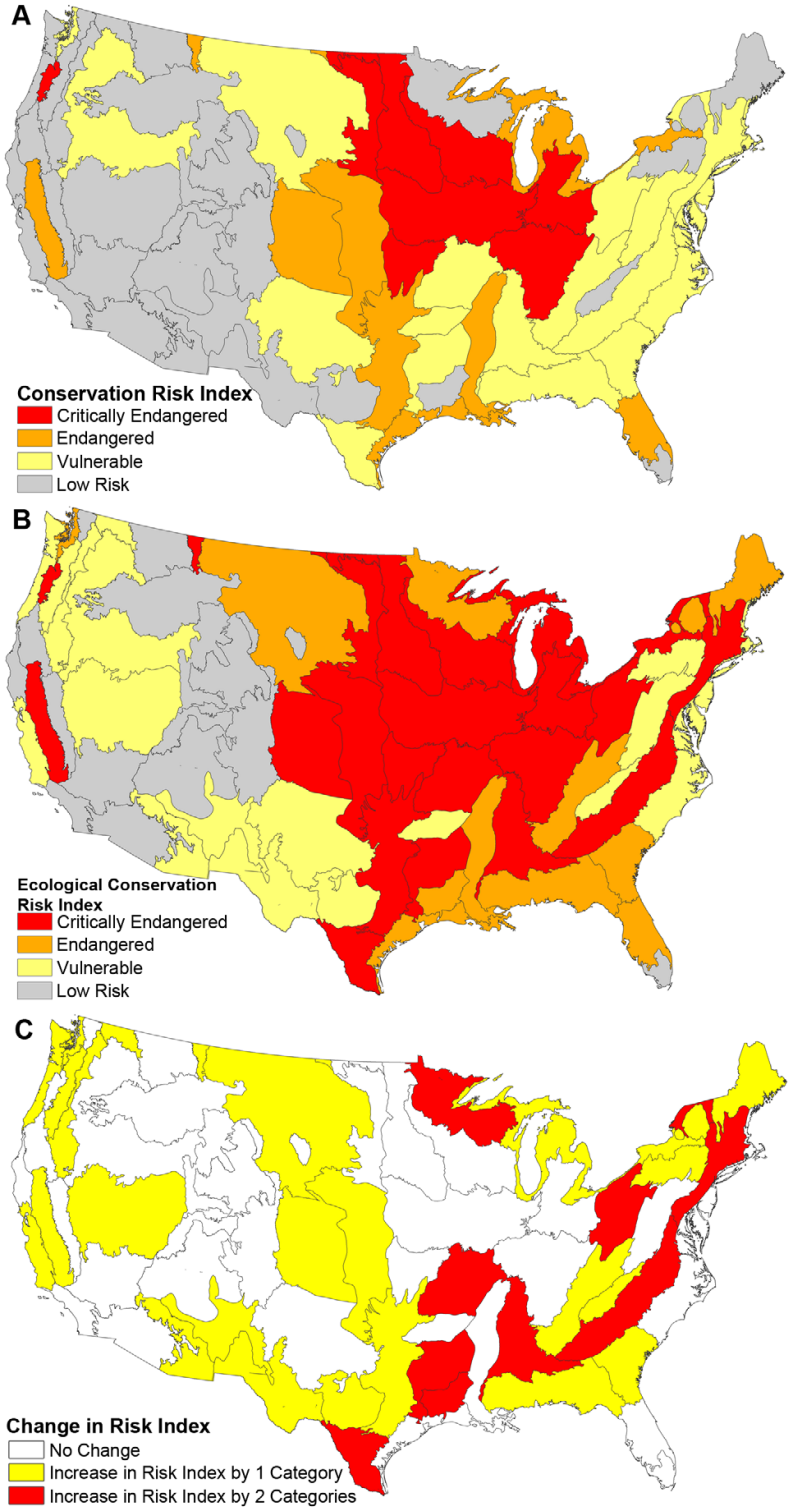
Ecological Conservation Risk Index shows increased risk for ecoregions compared to a Conservation Risk Index that does not include ecosystem alteration. (A) Conservation Risk Index, calculated following [19]. (B) Ecological Conservation Risk Index, which includes ecosystem alteration. (C) Increased risk measured by the Ecological Conservation Risk Index, quantified as the number of risk categories by which each ecoregion increased. Grey lines indicate ecoregional boundaries.

Based on the relationship between the amount of ecosystem conversion and the amount of land protection, the original Conservation Risk Index [19] identified 20 Vulnerable, 9 Endangered, and 9 Critically Endangered ecoregions across the United States (Fig. 3A). When we add in the new ecological alteration data, we find a dramatic increase in critically endangered ecoregions (from 9 to 23, with a range from 17 to 29 critically endangered ecosystems in our sensitivity analysis; Fig. 3B, C, and Table S1). Critically endangered areas included large areas of deciduous forest (from New England to Appalachia) and grasslands (in the central United States) with high levels of ecosystem alteration that went undetected using previous habitat conversion assessments. Overall, the inclusion of ecosystem alteration increased the conservation risk index across the United States such that 35 of the 69 ecoregions increased by one or two risk categories (Fig. 3B, C). The number of ecoregions with increased risk ranged from 22 to 44 in our sensitivity analysis.

## Discussion

Our ecological conservation risk assessment (ECRI) reveals ecoregions to be at greater risk than was apparent based on land conversion alone. Over half of the conterminous United States is either converted or highly altered. However, these impacts are not evenly distributed, with some ecoregions receiving a disproportionate share of ecosystem alteration and conversion. Notably, the three ecoregions with the highest percent of land that was highly altered were the Northern Appalachians, West Gulf Coastal Plain, and Southern Blue Ridge. While the vegetation in the Northern Appalachian and Southern Blue Ridge Mountains is only 4–11% converted to row crop or urban uses, current vegetation lacks the tall closed-canopy characteristics of the old growth forests that historically dominated these areas. In the West Gulf Coastal Plain, vegetation has shifted from Wet Longleaf Pine Savanna and Flatwoods (33% of the ecoregion historically) and Upland Longleaf Pine Forest and Woodland (22% of the ecoregion historically) vegetation to 23% uncharacteristic vegetation cover, primarily Loblolly pine (*Pinus taeda*) plantations. Taking this ecosystem alteration into account increased the assessed conservation risk to these ecoregions, elevating them to Vulnerable or Endangered status. In total, consideration of ecosystem alteration caused 35 ecoregions to increase one or two risk levels. This highlights the need for significant conservation efforts focused on sustainable vegetation management and landscape-scale vegetation restoration to reduce conservation risk.

**Figure 4:**
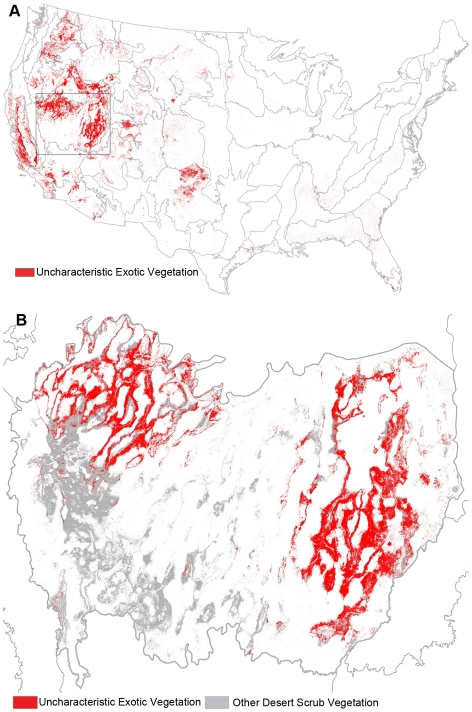
Uncharacteristic exotic vegetation in (A) the United States and (B) the Great Basin ecoregion. The area bordered by a dotted line in panel (A) is magnified in panel (B). Vegetation that is unique when compared to pre-settlement reference conditions is considered uncharacteristic. Uncharacteristic vegetation can be generated by either native or exotic vegetation; here we show the areas dominated by exotic vegetation. Grey lines indicate ecoregional boundaries.

Ecosystem alteration can be addressed with improved land management, using management actions that are targeted to the causes of ecosystem alteration. The proximate causes of alteration are characterized by the LANDFIRE ecosystem alteration dataset, which indentifies areas that have altered canopy cover, canopy height, or species composition. Loss of old growth, such as via logging, can be detected by reductions in canopy height and cover and shifts in species composition. Increases in canopy cover and shifts in composition can indicate fire suppression. And increases in “exotic uncharacteristic vegetation” explicitly identify areas that have been invaded by exotic plants (Fig. 4). These signatures of logging, fire suppression, and invasive species provide a national overview of the need for forest protection and improved forestry techniques to restore old growth forest characteristics, prescribed fire to restore natural fire regimes, and regionally specific approaches, such as appropriate grazing practices, to fight invasive species. We illustrate this with three examples: 1) Great Basin Desert Scrub [32], 2) Ozarks Oak Woodland [33], and 3) Western Cascades Western Hemlock Forest [34].

**Figure 5:**
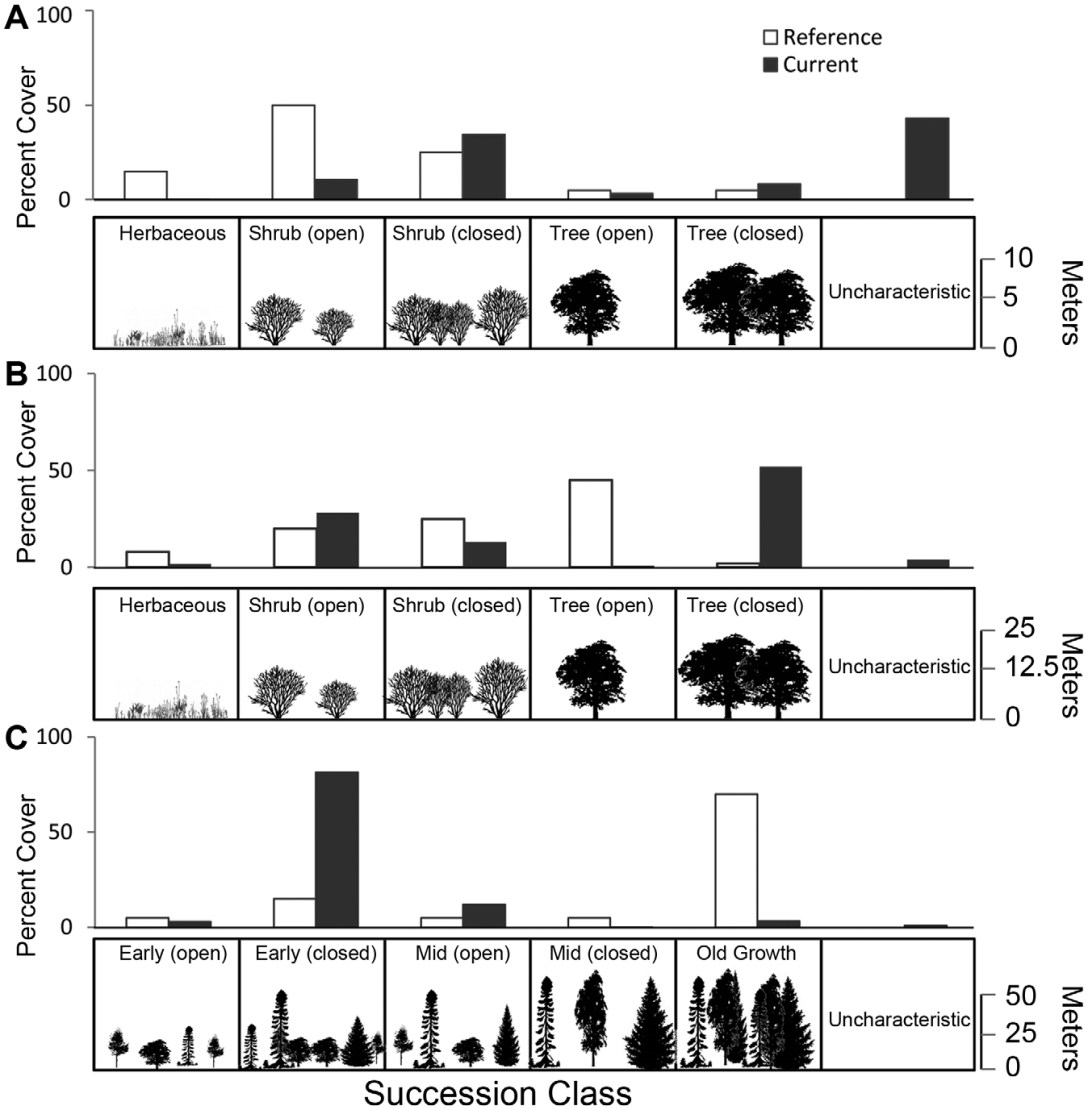
Current and reference successional classes for three ecosystems. Departure from reference conditions can be caused by (A) increases in uncharacteristic vegetation, as in Great Basin Salt Desert Scrub, (B) increases in closed canopy successional classes, as in Ozark Oak Woodland, or (C) increases in early successional classes, as in Cascades Western Hemlock Forest. These vegetation changes are the expected outcomes of biological invasion, fire suppression, and logging, respectively.

In the Great Basin, invasive species are a leading cause of ecological alteration (Fig. 5A; Fig. 4). Currently, over 25% of the Great Basin Desert Scrub ecosystem is mapped as “Uncharacteristic Exotic” in LANDFIRE (Fig. 4B), presumably due to the invasion of cheatgrass (*Bromus tectorum*), estimated to cover 20,000 km^2^ [35]. In the Ozarks Oak Woodlands, fire suppression is a leading cause of ecosystem alteration (Fig. 5B). The Ozarks Oak Woodland ecosystem currently exhibits mostly closed canopy conditions (∼80% of land cover) that were less common under reference conditions (∼20% of land cover) due to relatively frequent low intensity surface fires across the ecosystem prior to significant European settlement [36], [37]. In Western Hemlock Forests of the Western Cascades, logging is a leading cause of ecological alteration (Fig. 5C). Under reference conditions, these Western Hemlock Forests were dominated by tall (>50 m), closed canopy, old growth Douglas fir (*Pseudotsuga menziesii*) and western hemlock (*Tsuga heterophylla*) stands (∼70% of land cover). Currently, however, the landscape is dominated by closed canopy young forest stands 5–50 m tall (∼82% of land cover), a result of decades of logging [38].

In all three cases, biodiversity conservation is threatened by ecosystem alteration. Cheatgrass invasion of Desert Scrub threatens species including sage grouse (*Centrocercus urophasianus*) and desert tortoise (*Gopherus agassizii*) [39], [40]. Fire suppression in the Ozarks threatens savanna-dependent species such as the eastern collared lizard (*Crotaphytus collaris collaris*) [41]. Loss of Western Hemlock old growth forest threatens bird species such as the Marbled Murrelet (*Brachyramphus marmoratus*) [42], mammals such as northern flying squirrels (*Glaucomys sabrinus*) [43], and ectomycorrhizal fungi unique to forests with old-growth characteristics [44].

Although protected areas generally provide abatement from some threats to biodiversity such as development and forest clearing, we found that even within protected areas, 21% of non-converted lands have high levels of ecosystem alteration. This finding suggests that increased attention to management or restoration of vegetation conditions on our public lands is warranted. For example, to address widespread fire suppression in fire-dependant forests, some level of fire regime restoration and fuels treatment will be needed for restoration of both biodiversity and ecosystem services such as carbon storage [45], [46], [47]. Fire suppression can lead to increased risk of costly catastrophic fires in many ecosystems [48], [49] often due to an unnatural buildup of fuels [50], [51]. Ongoing large-scale federal efforts such as Landscape Conservation Cooperatives and US Forest Service forest plan revisions could benefit from the ecosystem alteration information presented here to both assess the need for restoration and to help target management activities. Restoration and management of vegetation within public protected areas may be more feasible than on private lands, which commonly have smaller parcel sizes and typically lack mechanisms for coordinating management across parcels.

With the addition of an ecological alteration dataset to the original Conservation Risk Index based only on land conversion, our analysis provides a more complete picture of the conservation status of ecoregions and can help identify not only areas in need of greater protection, but also areas in need of improved land management. While important, land protection strategies alone will be insufficient to meet conservation risks that we have identified. Successful conservation strategies will also require broader application of ecologically based vegetation management such as: 1) restoration of fire regimes and/or increased use of fire surrogates, 2) forestry techniques that accelerate development of appropriate vegetation structure and composition, 3) invasive species control, and 4) improved grazing practices. Greater resources should be directed to ecosystem management, particularly within the ecoregions at greatest conservation risk as a result of ecosystem alteration.

## Supporting Information

Table S1 Ecosystem conversion and alteration in the ecoregions of the United States.
(DOCX)
