# Supplementary material for: Accounting for Ecosystem Alteration Doubles Estimates of Conservation Risk in the Conterminous United States
Source: PLoS One. 2011 Aug 5;6(8):e23002. doi: 10.1371/journal.pone.0023002 (PMC3151280; doi:10.1371/journal.pone.0023002)
Supplement: Table S1 — Ecosystem conversion and alteration in the ecoregions of the United States. (DOCX) [file pone.0023002.s001.docx]

Table S1. Ecosystem conversion and alteration in the ecoregions of the United States.

| Major Habitat Type^1^ | Ecoregion | Protected (%) | Highly Altered (%) | Converted (%) | Agriculture (%) | Urban (%) | Crisis Ecoregions (CRI) | Crisis Ecoregions (ECRI) |
| --- | --- | --- | --- | --- | --- | --- | --- | --- |
| Deserts and Xeric Shrublands | |  |  |  |  |  |  |  |
|  | Apache Highlands* | 10.54 | 33.16 | 1.29 | 0.67 | 0.63 |  | Vulnerable |
|  | Chihuahuan Desert* | 5.89 | 49.39 | 1.78 | 0.98 | 0.80 |  | Vulnerable |
|  | Colorado Plateau | 19.31 | 12.48 | 2.26 | 1.62 | 0.64 |  |  |
|  | Columbia Plateau | 9.46 | 19.78 | 22.06 | 20.03 | 2.03 | Vulnerable | Vulnerable |
|  | Great Basin | 8.02 | 25.99 | 3.60 | 2.34 | 1.26 |  | Vulnerable |
|  | Mojave Desert | 43.51 | 4.73 | 1.74 | 0.26 | 1.47 |  |  |
|  | Sonoran Desert* | 26.29 | 4.11 | 8.28 | 5.52 | 2.77 |  |  |
|  | Tamaulipan Thorn Scrub* | 0.23 | 35.16 | 22.79 | 17.24 | 5.55 | Vulnerable | Critically Endangered |
|  | Wyoming Basins | 1.80 | 27.02 | 4.77 | 3.98 | 0.79 |  | Vulnerable |
| Flooded Grasslands and Savannas | |  |  |  |  |  |  |  |
|  | Tropical Florida | 51.72 | 11.34 | 28.22 | 16.67 | 11.54 |  |  |
| Mediterranean Forests, Woodlands and Scrub | |  |  |  |  |  |  |  |
|  | California Central Coast | 9.99 | 35.93 | 12.78 | 6.72 | 6.06 |  | Vulnerable |
|  | California South Coast* | 19.08 | 15.87 | 18.05 | 2.16 | 15.89 |  |  |
|  | Great Central Valley | 2.34 | 13.84 | 49.74 | 43.33 | 6.41 | Endangered | Critically Endangered |
| Temperate Broadleaf and Mixed Forests | |  |  |  |  |  |  |  |
|  | Central Appalachian Forest | 9.97 | 37.26 | 30.26 | 21.78 | 8.48 | Vulnerable | Vulnerable |
|  | Chesapeake Bay Lowlands | 5.95 | 16.10 | 40.22 | 30.48 | 9.75 | Vulnerable | Vulnerable |
|  | Cumberlands and Southern Ridge and Valley | 2.92 | 38.56 | 25.77 | 17.23 | 8.53 | Vulnerable | Endangered |
|  | Great Lakes* | 2.53 | 21.68 | 41.73 | 29.97 | 11.76 | Endangered | Critically Endangered |
|  | High Allegheny Plateau | 11.58 | 43.22 | 22.56 | 18.26 | 4.30 |  | Vulnerable |
|  | Interior Low Plateau | 1.55 | 21.10 | 53.47 | 45.40 | 8.07 | Critically Endangered | Critically Endangered |
|  | Lower New England / Northern Piedmont | 2.39 | 39.87 | 34.05 | 18.70 | 15.36 | Vulnerable | Critically Endangered |
|  | Mississippi River Alluvial Plain | 5.25 | 1.85 | 68.37 | 63.18 | 5.19 | Endangered | Endangered |
|  | North Atlantic Coast | 9.30 | 23.64 | 41.21 | 10.87 | 30.34 | Vulnerable | Vulnerable |
|  | North Central Tillplain | 0.90 | 6.02 | 81.04 | 67.14 | 13.91 | Critically Endangered | Critically Endangered |
|  | Northern Appalachian / Acadian* | 4.24 | 74.77 | 6.42 | 4.08 | 2.34 |  | Endangered |
|  | Ouachita Mountains | 3.20 | 11.22 | 25.86 | 21.25 | 4.60 | Vulnerable | Vulnerable |
|  | Ozarks | 2.02 | 43.30 | 37.41 | 31.58 | 5.84 | Vulnerable | Critically Endangered |
|  | Piedmont | 0.87 | 63.13 | 31.21 | 20.06 | 11.15 | Vulnerable | Critically Endangered |
|  | Prairie-Forest Border | 2.52 | 12.42 | 70.34 | 61.29 | 9.04 | Critically Endangered | Critically Endangered |
|  | Southern Blue Ridge | 11.20 | 70.15 | 15.55 | 10.38 | 5.17 |  | Vulnerable |
|  | St. Lawrence - Champlain Valley* | 0.68 | 23.91 | 36.43 | 30.49 | 5.94 | Vulnerable | Critically Endangered |
|  | Superior Mixed Forest* | 5.60 | 42.01 | 15.89 | 12.66 | 3.23 |  | Endangered |
|  | Upper East Gulf Coastal Plain | 1.27 | 33.96 | 35.34 | 28.35 | 6.99 | Vulnerable | Critically Endangered |
|  | Western Allegheny Plateau | 2.44 | 40.38 | 36.15 | 24.18 | 11.97 | Vulnerable | Critically Endangered |
|  | Willamette Valley - Puget Trough - Georgia Basin: Temperate Broadleaf and Mixed Forests | 0.93 | 13.39 | 61.77 | 51.70 | 10.07 | Critically Endangered | Critically Endangered |
| Temperate Conifer Forests | |  |  |  |  |  |  |  |
|  | Arizona-New Mexico Mountains | 8.80 | 40.15 | 1.44 | 0.47 | 0.97 |  | Vulnerable |
|  | Black Hills | 3.53 | 13.54 | 2.90 | 1.44 | 1.46 |  |  |
|  | California North Coast | 6.06 | 1.58 | 1.78 | 1.10 | 0.69 |  |  |
|  | Canadian Rocky Mountains* | 15.66 | 8.48 | 4.47 | 3.86 | 0.61 |  |  |
|  | East Cascades - Modoc Plateau | 8.69 | 28.74 | 6.12 | 5.33 | 0.79 |  | Vulnerable |
|  | East Gulf Coastal Plain | 1.91 | 23.70 | 24.48 | 17.74 | 6.74 | Vulnerable | Endangered |
|  | Florida Peninsula | 3.14 | 12.97 | 44.11 | 27.35 | 16.76 | Endangered | Endangered |
|  | Klamath Mountains | 13.59 | 12.63 | 4.86 | 3.95 | 0.92 |  |  |
|  | Mid-Atlantic Coastal Plain | 5.70 | 22.32 | 31.56 | 24.51 | 7.05 | Vulnerable | Vulnerable |
|  | Middle Rockies - Blue Mountains | 15.74 | 13.04 | 5.06 | 4.66 | 0.41 |  |  |
|  | North Cascades* | 42.81 | 11.48 | 0.78 | 0.56 | 0.23 |  |  |
|  | Okanagan* | 11.64 | 26.07 | 5.34 | 3.17 | 2.16 |  | Vulnerable |
|  | Pacific Northwest Coast* | 13.34 | 24.87 | 3.01 | 2.42 | 0.59 |  | Vulnerable |
|  | Sierra Nevada | 27.63 | 9.80 | 1.23 | 0.50 | 0.73 |  |  |
|  | South Atlantic Coastal Plain | 4.23 | 21.86 | 22.58 | 15.04 | 7.54 | Vulnerable | Endangered |
|  | Southern Rocky Mountains | 15.13 | 19.54 | 5.28 | 4.35 | 0.93 |  |  |
|  | Upper West Gulf Coastal Plain | 1.06 | 57.04 | 23.72 | 18.06 | 5.66 | Vulnerable | Critically Endangered |
|  | Utah High Plateaus | 9.25 | 13.84 | 2.72 | 1.53 | 1.19 |  |  |
|  | Utah-Wyoming Rocky Mountains | 30.23 | 6.83 | 4.21 | 3.51 | 0.70 |  |  |
|  | West Cascades | 16.26 | 46.87 | 0.79 | 0.54 | 0.24 |  | Vulnerable |
|  | West Gulf Coastal Plain | 7.04 | 71.03 | 10.64 | 6.81 | 3.83 |  | Endangered |
|  | Willamette Valley - Puget Trough - Georgia Basin: Temperate Conifer Forests* | 1.14 | 24.15 | 23.06 | 13.07 | 9.99 | Vulnerable | Endangered |
| Temperate Grasslands, Savannas and Shrublands | |  |  |  |  |  |  |  |
|  | Aspen Parkland* | 2.56 | 9.39 | 82.83 | 78.17 | 4.66 | Critically Endangered | Critically Endangered |
|  | Central Mixed-Grass Prairie | 0.69 | 14.43 | 41.77 | 37.98 | 3.79 | Endangered | Critically Endangered |
|  | Central Shortgrass Prairie | 0.89 | 9.92 | 41.13 | 37.21 | 3.92 | Endangered | Critically Endangered |
|  | Central Tallgrass Prairie | 0.59 | 8.72 | 81.05 | 73.60 | 7.45 | Critically Endangered | Critically Endangered |
|  | Crosstimbers and Southern Tallgrass Prairie | 0.88 | 21.89 | 40.72 | 31.94 | 8.78 | Endangered | Critically Endangered |
|  | Dakota Mixed-Grass Prairie | 0.99 | 5.98 | 69.45 | 65.38 | 4.07 | Critically Endangered | Critically Endangered |
|  | Edwards Plateau | 0.40 | 29.38 | 4.19 | 2.04 | 2.15 |  | Vulnerable |
|  | Fescue-Mixed Grass Prairie* | 1.38 | 4.81 | 46.56 | 44.67 | 1.89 | Endangered | Critically Endangered |
|  | Northern Great Plains Steppe* | 1.86 | 18.99 | 26.05 | 24.27 | 1.78 | Vulnerable | Endangered |
|  | Northern Tallgrass Prairie* | 1.48 | 1.37 | 88.30 | 82.15 | 6.14 | Critically Endangered | Critically Endangered |
|  | Osage Plains/Flint Hills Prairie | 0.92 | 12.74 | 51.41 | 44.88 | 6.53 | Critically Endangered | Critically Endangered |
|  | Southern Shortgrass Prairie | 0.85 | 18.75 | 20.83 | 18.36 | 2.48 | Vulnerable | Vulnerable |
| Tropical and Subtropical Grasslands, Savannas and Shrublands | | |  |  |  |  |  |  |
|  | Gulf Coast Prairies and Marshes* | 4.65 | 17.14 | 49.91 | 39.76 | 10.15 | Endangered | Endangered |
| ^1^The Nature Conservancy. Unpublished Material. Sep 2006. Terrestrial Ecoregions. Vector digital data. Arlington, VA. The Nature Conservancy. | | | | | | | | |
| *Ecoregion crosses the international boundary; results shown for the portion of the ecoregion in the conterminous US. | | | | | |  |  |  |
